# A Comparison of the Gluco-Regulatory Responses to High-Intensity Interval Exercise and Resistance Exercise

**DOI:** 10.3390/ijerph18010287

**Published:** 2021-01-02

**Authors:** Brett A. Gordon, Caroline J. Taylor, Jarrod E. Church, Stephen D. Cousins

**Affiliations:** 1 Holsworth Research Initiative, La Trobe Rural Health School, La Trobe University, Bendigo, VIC 3550, Australia; stephen.cousins@latrobe.edu.au; 2 Department of Physiology, Anatomy & Microbiology, La Trobe University, Bundoora, VIC 3086, Australia; Caroline.Taylor@latrobe.edu.au (C.J.T.); j.church@latrobe.edu.au (J.E.C.)

**Keywords:** resistance training, high-intensity interval training, metabolism, insulin, stress, exercise

## Abstract

High-intensity interval exercise and resistance exercise both effectively lower blood glucose; however, it is not clear whether different regulatory mechanisms exist. This randomised cross-over study compared the acute gluco-regulatory and the physiological responses of high-intensity interval exercise and resistance exercise. Sixteen (eight males and eight females) recreationally active individuals, aged (mean ± SD) 22 ± 7 years, participated with a seven-day period between interventions. The high-intensity interval exercise trial consisted of twelve, 30 s cycling intervals at 80% of peak power capacity and 90 s active recovery. The resistance exercise trial consisted of four sets of 10 repetitions for three lower-limb exercises at 80% 1-RM, matched for duration of high-intensity interval exercise. Exercise was performed after an overnight fast, with blood samples collected every 30 min, for two hours after exercise. There was a significant interaction between time and intervention for glucose (*p* = 0.02), which was, on average (mean ± SD), 0.7 ± 0.7 mmol∙L^−1^ higher following high-intensity interval exercise, as compared to resistance exercise. Cortisol concentration over time was affected by intervention (*p* = 0.03), with cortisol 70 ± 103 ng∙mL^−1^ higher (*p* = 0.015), on average, following high-intensity interval exercise. Resistance exercise did not induce the acute rise in glucose that was induced by high-intensity interval exercise and appears to be an appropriate alternative to positively regulate blood glucose.

## 1. Introduction

Appropriate regulation of blood glucose is important for the maintenance of health and prevention of chronic disease states, such as diabetes and cardiovascular disease. High-intensity interval exercise is purported to be a time-efficient exercise modality that improves blood glucose control after a period of training [1]. High-intensity interval exercise is also suggested to be more enjoyable than other aerobic exercise modalities, such as moderate-intensity continuous training, and might therefore lead to improved exercise compliance [2,3], leading to improved glucose regulation. Despite the completion of resistance training being demonstrated to reduce the likelihood of impaired glucose metabolism [4], it has not been widely reported as either time-efficient or enjoyable. Even though both high-intensity interval exercise and resistance exercise modalities commonly involve repeated short bouts of vigorous exercise, followed by a period of low-intensity exercise or rest [5,6], it is not clear if similar acute gluco-regulatory responses occur. High-intensity interval exercise typically requires the use of expensive equipment or high-impact activities and therefore might be less appealing or feasible than low-impact resistance exercise, which can be completed with or without specialised equipment. If resistance exercise is demonstrated to be at least as effective as high-intensity interval exercise for regulating blood glucose immediately after exercise, this could have important implications for the prescription of exercise for the maintenance of health.

Resistance or high-intensity sprint interval exercise matched for exercise duration has resulted in similar improvements to glucose tolerance in young, insufficiently active males, 12 h after exercise [7]. In comparison to no exercise, high-intensity interval training can reduce post-prandial hyperglycaemia [8]. However, high-intensity exercise can cause a marked rise in glucose production [9] and post-exercise hyperglycaemia and hyperinsulinaemia for up to 60 min [10]. Over time, hyperglycaemia can have negative consequences on tissues and organs, such as the kidneys, eyes and nerves, and should therefore be avoided where possible [11]. It is possible the associated transient hyperinsulinaemia can lead to pancreatic β-cell abuse and reduced metabolic control [12]. Hyperglycaemia resulting from stress has been associated with elevated cortisol concentrations [13], and higher-intensity exercise of both aerobic [14] and resistance [15] modalities results in higher cortisol concentrations. It is therefore likely that glucose tolerance in response to exercise is influenced through a stress response measured by cortisol. Cortisol might also be responsible for determining which energy system is used and substrate utilised through influencing the catabolising of amino-acids into free fatty acids (FFA) and the rate of gluconeogenesis [14].

Initial efforts in a high-intensity interval exercise bout have an almost equal contribution from anaerobic and aerobic energy sources; however, later efforts of the bout are largely reliant on aerobic energy provision [16]. In contrast, a single bout of resistance exercise appears to be largely reliant on anaerobic energy metabolism [17]. However, it appears that, when high-intensity interval exercise and resistance exercise are matched for both duration and effort, contributions from both aerobic and anaerobic energy systems are similar [18]. Energy expenditure is associated with changes to blood glucose, and the energy system used during exercise will determine hormonal and physiological change. Time spent exercising and the intensity of exercise are likely to be critical factors for glucose regulation, as when total duration but not work time or intensity are matched, differences in energy expenditure are observed [19]. It is possible that energy expenditure as a result of exercise is responsible for changes in glucose and insulin with a negative association observed for both [20], but this has only been investigated in continuous aerobic exercise and not interval exercise or resistance exercise. It is not appropriate to assume that the responses from continuous aerobic exercise will be similar to those experienced after interval- or resistance-based exercise.

Although a comparison of interval exercise and resistance exercise has occurred [7], an adequate investigation of different exercise modes (aerobic interval and resistance exercise) involving high-intensity work efforts interspersed with rest/recovery periods has not been conducted. As a proof of concept, it is important to determine if interval exercise or resistance exercise infers different gluco-regulatory responses and if this is as a result of physiological differences. Previous research suggests a varied gluco-regulatory hormonal response to each exercise modality, due to expected differences in energy-system use [19], despite any similarity in overall energy expenditure [18]. However, the lack of investigation of work-matched modes of high-intensity exercise prevents the clarity of this. Identifying whether differences in glucose regulation exist and determining if they are related to different physiological responses between modes of exercise will have important implications for optimising exercise prescription. Therefore, the primary aim of this study was to compare the acute gluco-regulatory (glucose, insulin and cortisol) and the physiological (heart rate, rate of perceived exertion (RPE), lactate and FFA) response to different modes of exercise: high-intensity interval exercise and resistance exercise in young, healthy individuals.

## 2. Materials and Methods

### 2.1. Design

**Figure 1:**
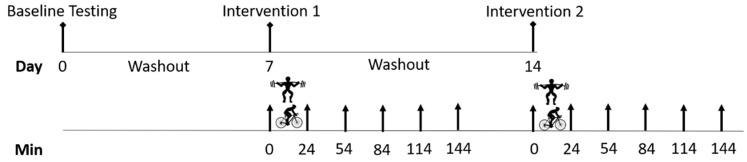
Study protocol schematic. The symbol 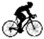 = high-intensity cycling exercise; 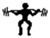 = resistance exercise; 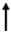 = blood sample; Min = minutes. The high-intensity cycling exercise and resistance exercise were completed in a random order, with venous blood samples collected immediately before and after exercise, and then every 30 min, for two hours.

This study used a randomised cross-over trial design to compare the effect of high-intensity interval exercise and resistance exercise on glucose regulation in healthy adults. A group of recreationally trained adults participated in a single bout of interval exercise and a single bout of resistance exercise prescribed at the same relative high intensity (80% of capacity), seven days apart. Exercise bouts were performed in the morning, between 7:00 a.m. and 9:00 a.m., and all participants completed both trials at the same time of day. Plasma glucose, insulin and cortisol levels were evaluated before, immediately, 30, 60, 90 and 120 min after each exercise bout, in order to compare the acute gluco-regulatory response to each exercise modality. Physiological responses of heart rate and rate of perceived exertion (RPE) were recorded after each work interval, during both interventions with blood lactate and plasma FFA concentrations evaluated up to two hours after each exercise bout. The timing of the protocol is indicated in Figure 1.

### 2.2. Participants

Individuals were eligible to participate in this study if they were aged 18 to 50 years, apparently healthy, had no recent history of injuries and regularly (≥twice per week) completed both high-intensity interval-based and resistance-based exercise. Individuals were excluded if they had current or prior musculoskeletal injuries that would be exacerbated by exercise. After a full explanation of study procedures, participants were informed of the benefits and risks of the investigation and provided written informed consent to participate in the study, which was approved by the University’s Human Ethics Committee (14-041). Sixteen individuals (8 males and 8 females) met the eligibility criteria and volunteered to participate.

### 2.3. Procedures

Prior to completing initial testing procedures, all participants were familiarised with the cycle ergometer and testing protocols, including evaluation of proper exercise technique on each testing exercise, and any questions they had were answered. Participants then attended two preliminary visits, where their physical characteristics were measured, and assessments of cardiovascular capacity and muscular strength were completed. Cardiovascular capacity was assessed by modifying the protocol described by Hawley and Noakes [21] to predict peak power output, with the initial workload reduced to 1.5 W∙kg^−1^ of body mass. The assessment was conducted by using a Lode cycle ergometer (Sport Excalibur, the Netherlands), maintaining a cadence of 70 rpm. Workloads were increased every 150 s by 25 W, until voluntary exhaustion. Voluntary exhaustion was considered when participants were unable to maintain a cadence of 70 rpm or, despite encouragement, did not want to continue. From this, peak power output (*W_peak_*) was determined by using the following equation:

$$W_{peak}\left(W\right)\;=\;W_{final}+\;\left(\frac{t}{150}\;\times \;25\right)$$

where *W_final_* is the workload of the final stage, and *t* is the completed duration in seconds of the final stage. We were then able to estimate *VO*_2*peak*_ by using the following equation:

$$VO_{2peak}\;\left(L\cdot \min^{-1}\right)\;=\;0.01141\;\times \;W_{peak}\;\left(W\right)\;+\;0.435$$

*VO*_2*peak*_ was then converted to mL∙min^−1^ and made relative to body mass. Muscular strength was assessed via 3-repetition maximum (3-RM) on the movements to be used in the resistance exercise protocol: squats, calf raise and incline leg press (conducted in that order). Before attempting a 3-RM, participants performed 1 set of 7 and then 5 repetitions, respectively, with light loads (≤30% 1-RM and 50% 1-RM, respectively), and then 3 repetitions with an increasing heavier load. If the exercise was performed with correct technique, the weight was increased by 2.5–10 kg. The increments in weight were dependent upon the effort required for the lift and became progressively smaller as the participant approached the 3-RM. Failure was defined as an exercise falling short of the full range of motion on at least 2 attempts spaced at least 2 min apart. The Brzycki method was used to predict 1-RM from the absolute weight lifted and the number of repetitions that were correctly completed [22] for the determination of exercise load.

Following the completion of preliminary testing, the order of the two trials (high-intensity interval exercise and resistance exercise) was randomly allocated by an independent investigator who generated a random sequence (using www.randomization.com) and secured each sequence in an opaque envelope. Each envelope was opened sequentially. At least seven days separated the final preliminary testing session and the first exercise trial and between the first and second exercise trials [7,19]. This was to minimise any potential remaining physiological effect of the previous exercise bout. Participants were required to attend the laboratory for each exercise trial after a minimum eight-hour fast and without completing any strenuous activity for at least 72 h, which was confirmed verbally. Diet was not controlled beyond requiring the eight-hour fast; however participants were asked to replicate their diet in the three days prior to each protocol, which was confirmed by comparing 3-day food diaries completed by participants.

### 2.4. Exercise Trials

Participants remained fasted throughout the trial, with water allowed ad libitum. The high-intensity interval exercise (HIE) trial involved participants completing twelve 30 s bouts of cycling at 80% of their predicted peak power on a Lode cycle ergometer (Excalibur Sport), followed by 90 s of very light intensity active recovery. The resistance exercise (RE) trial required participants to complete four sets of 10 repetitions at 80% of their predicted 1-RM for the three movements tested: squats, calf raise and incline leg press, utilising a two second concentric phase and one second eccentric phase (2:0:1:0) tempo. Participants were given 90 s recovery between sets. The HIE and RE trials were prescribed at the same relative exercise intensity (80% of capacity), with matched duration, working similar muscles. Heart rate (monitored using a Polar™ monitor) and RPE, using Borg’s 6–20 scale (which all participants were familiar with), were recorded after each work interval during both trials, with heart rate reported as a percentage of their age-predicted maximum.

### 2.5. Biochemistry

Prior to each exercise trial, a catheter was inserted into an antecubital vein, to allow for blood sampling. Blood samples were collected immediately prior to exercise, along with immediately, and 30, 60, 90 and 120 min following exercise. Samples were collected in EDTA containing vacutainer tubes. Aliquots of whole blood were analysed for lactate, using the YSI 2900 StatPlus biochemistry analyser (YSI Inc., Yellow Springs, OH, USA). Patency of the catheter was maintained by injecting saline after each blood collection, with the first 3 mL of each sample discarded, to ensure blood samples were not contaminated. Plasma was separated by centrifugation at 1000 rpm and 4 °C, with plasma stored in multiple aliquots at −80 °C for later batch analysis. Plasma glucose was determined by using the YSI 2900 StatPlus biochemistry analyser. Plasma insulin was determined by using Quantikine^®^ ELISA kits (R&D Systems) (In Vitro Technologies Pty. LTD., Noble Park, VIC, Australia) for Human/Canine/Porcine Insulin. Plasma cortisol and FFA concentrations were determined by using a cortisol ELISA kit (Abcam, Melbourne, VIC, Australia) and FFA quantification kit (Abcam, Melbourne, VIC, Australia), respectively. All kits were used according to manufacturer’s instructions, with samples measured in duplicate and the mean of the two outcomes reported. If there was greater than 5% variation between the duplicate samples, the individual sample was re-analysed in duplicate.

### 2.6. Statistical Analysis

To be able to effectively calculate sample size, we conducted a pilot with three participants, comparing the glucose response to HIE and RE, which identified a mean difference across five time-points between HIE and RE of 0.4 ± 0.8 mmol∙L^−1^, with an effect size of 0.37. Using this conservative estimate of effect and a repeated measures mixed-model ANOVA design for two groups with five repeat measures (post exercise) and 95% power at an alpha level of 0.05, we required 16 participants.

After confirming normal distribution of the data by using the Shapiro–Wilk statistic and visual assessment, two-way mixed-model (trial × time post-exercise) repeated measures ANOVA were conducted for glucose, lactate, insulin, cortisol and FFA, using IBM SPSS version 26 (IBM Corp., Armonk, NY, USA) for Windows. Where a significant interaction effect was identified, post hoc paired samples t-tests were conducted to identify differences between trials at individual time-points, with Bonferroni corrections made to account for multiple comparisons. To ensure the random order was successful, for significant interactions between trial and time, the trial order was included as an additional between factors variable. Additional trial by time mixed-model repeated measures ANOVA were conducted to assess for differences in heart rate (%HRmax) and RPE. In instances where the assumption of sphericity was violated, the Greenhouse–Geisser correction was applied. Missing data due to small plasma collections were estimated conservatively by bringing the last known data point forward, this occurred for 3 or 4 samples (approximately 2% of all samples) for insulin, cortisol and FFA. Data are reported as mean ± SD or mean difference (95% confidence interval). Results were considered statistically significant if *p* < 0.05. Effect sizes are reported as partial eta-squared (η_p_^2^), where <0.06 is considered a small change; 0.06 to 0.14 is a moderate change, and ≥0.14 is a large change.

## 3. Results

Participant characteristics are described in Table 1. There was no difference in glucose, insulin, cortisol, FFA or heart rate before either of the exercise trials; however, blood lactate was 0.53 (0.22 to 0.84) mmol∙L^−1^ higher before the RE trial than the HIE trial (t = 3.6_(15)_, *p* = 0.002; Table 2). Males were taller, heavier and had greater exercise capacity than females, except when oxygen uptake was expressed relative to body mass (Table 1).

**Figure 2:**
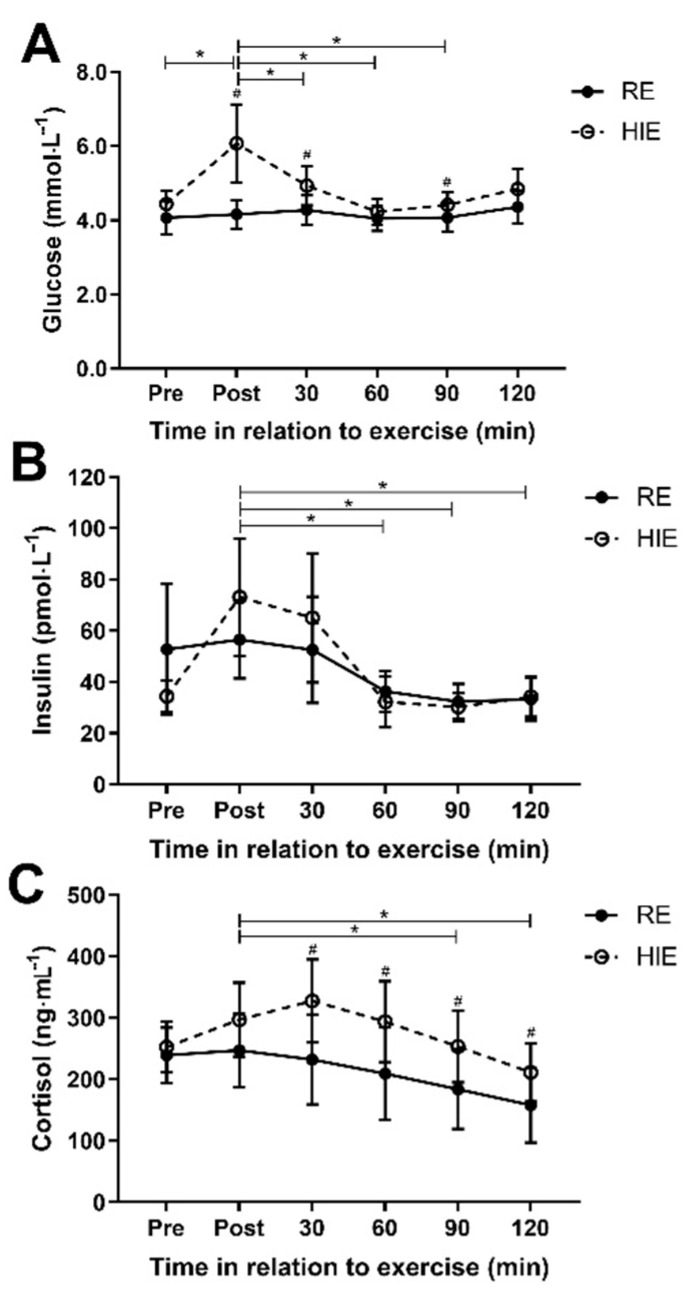
Responses to high-intensity interval exercise (HIE) and resistance exercises (RE) for plasma glucose (**A**), plasma insulin (**B**) and plasma cortisol (**C**). Data shown are mean and 95% CI. ^#^ Indicates a post hoc difference between exercise mode after a significant interaction effect was identified (*p* < 0.05). * Indicates a significant pairwise comparison after Bonferroni correction (*p* < 0.05) tested after a significant main effect for time was identified. Time of measurements were immediately pre- and post-exercise, and then 30, 60, 90 and 120 min after the completion of exercise.

There was a difference in the pattern of glucose response (interaction effect; Figure 2A) after exercise between HIE and RE (f = 4.6_(1.7)_, *p* = 0.026; η_p_^2^ = 0.23), with mean glucose concentration on average 0.7 (0.3 to 1.0) mmol∙l^−1^ higher with HIE (f = 11.0_(2.4)_, *p* < 0.001). Post hoc paired samples t-test identified significantly higher glucose concentrations following HIE immediately after exercise (1.9 (0.6 to 3.2); t = 3.2_(15)_, *p* = 0.006), 30 min after exercise (0.7 (0.1 to 1.2); t = 2.5_(15)_, *p* = 0.025) and 90 min after exercise (0.3 (0.02 to 0.7); t = 2.2_(15)_, *p* = 0.041). Trial order (f = 1.8_(1.8)_, *p* = 0.190; η_p_^2^ = 0.11) did not statistically affect the interaction between trial and time. There was no difference in the insulin response (f = 1.3_(1.7)_, *p* = 0.275; η_p_^2^ = 0.08; Figure 2B) to each trial. The pattern of cortisol response was different between HIE and RE (f = 3.4_(2.4)_, *p* = 0.036; η_p_^2^ = 0.19), with mean cortisol concentration 71 (16 to 126) ng∙mL^−1^ higher with HIE (f = 7.5_(1)_, *p* = 0.015; Figure 2C). Post hoc paired samples t-test identified significantly higher cortisol concentrations following HIE 30 min after exercise (95 (25 to 165); t = 2.9_(15)_, *p* = 0.011), 60 min after exercise (84 (21 to 148); t = 2.8_(15)_, *p* = 0.013), 90 min after exercise (70 (19 to 121); t = 2.9_(15)_, *p* = 0.011) and 120 min after exercise (54 (6 to 101); t = 2.4_(15)_, *p* = 0.030). The pattern of cortisol response was not statistically affected by trial order (f = 2.3_(2.3)_, *p* = 0.108; η_p_^2^ = 0.14).

**Figure 3:**
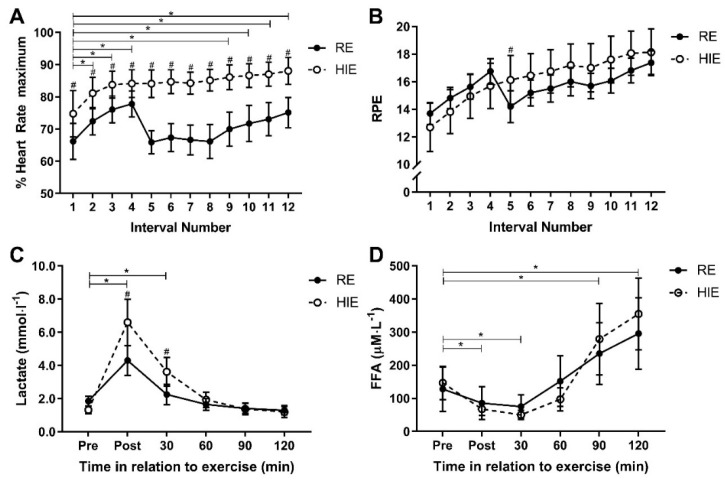
Responses to high-intensity interval exercise (HIE) and resistance exercises (RE) for % heart rate maximum (**A**), rating of perceived effort (**B**), blood lactate concentrations (**C**) and plasma free fatty acid concentrations (**D**). Data shown are mean and 95% CI. ^#^ Indicates a post hoc difference between exercise mode after a significant interaction effect was identified (*p* < 0.05). * Indicates a significant pairwise comparison after Bonferroni correction (*p* < 0.05) tested after a significant main effect for time was identified. For rate of perceived exertion (RPE), there were significant pairwise comparisons for time for all intervals in comparison to the first interval. Time of measurement for % heart rate maximum and RPE were at the completion of each work interval for both HIE and RE. Time of measurements for lactate and free fatty acids (FFA) were immediately pre- and post-exercise, and then 30, 60, 90 and 120 min after the completion of exercise.

A significant interaction effect for HR response (f = 11.2_(3.6)_, *p* < 0.001; η_p_^2^ = 0.45) and RPE response (f = 7.7_(3.1)_, *p* < 0.001; η_p_^2^ = 0.36) was present between trial and time. Mean exercise intensity, measured as %HRmax across the 12 intervals, was 13% (10% to 17%) higher (f = 67.2_(1)_, *p* < 0.001) with HIE (84% ± 7.4%), compared to RE (71% ± 7.5%), and %HRmax was higher following each HIE bout than each RE bout (*p* < 0.04; Figure 3A). However, mean exercise intensity measured by RPE, was similar (f = 0.3_(1)_, *p* = 0.605) for each HIE (16 ± 2.8) and RE (16 ± 1.3), with RPE only different between modes after the fifth work interval (1.9 (0.4 to 3.5); t = 2.7_(15)_, *p* = 0.016; Figure 3B). A significant interaction effect between trial and time for lactate (f = 11.8_(1.3)_, *p* = 0.001; η_p_^2^ = 0.44) suggested a difference in anaerobic energy utilisation (Figure 3C). Mean lactate concentration was 0.76 (0.20 to 1.33) mmol∙L^−1^ higher (f = 8.4_(1)_, *p* = 0.11) following HIE, as compared to RE. Lactate was higher following HIE, as compared to RE, immediately after exercise (2.30 (0.85 to 3.75); t = 3.4_(15)_, *p* = 0.004) and 30 min after exercise (1.37 (0.47 to 2.26); t = 3.3_(15)_, *p* = 0.005). The pattern of lactate response was not statistically affected by trial order (f = 3.4_(1.4)_, *p* = 0.067; η_p_^2^ = 0.20). There was no interaction between trial and time for FFA response (f = 3.0_(1.7)_, *p* = 0.076; η_p_^2^ = 0.17; Figure 3D).

## 4. Discussion

This study was designed to examine the acute gluco-regulatory and physiological responses to two different modes of exercise, high-intensity interval exercise and resistance exercise. In this sample of young healthy individuals, there was variation in the acute glucose response between the different modes of exercise. Glucose, cortisol and lactate concentrations were all higher after HIE than RE, while perception of effort during exercise and FFA concentration after exercise were similar with both HIE and RE. Resistance exercise was therefore demonstrated to be less likely to induce a transient increase in glucose than high-intensity interval exercise.

A rise in plasma glucose was observed in response to HIE that was similar to that previously observed [10]; however, RE did not induce this same rise. Meanwhile the insulin response was not different between HIE and RE. Although this does not suggest improved insulin sensitivity with RE, a reduced insulin response and greater insulin sensitivity with resistance exercise, in comparison to sprint interval exercise, has been demonstrated in inactive males approximately 12 h after the exercise bout [7]. Furthermore, a single bout of resistance exercise failed to change the overall glucose response to an oral glucose tolerance test (OGTT), in young healthy individuals, although the insulin response was reduced from pre-exercise [23]. With the known reduction in post-prandial hyperglycaemia following high-intensity interval exercise [8], the findings from the current study provide evidence of a potential role for resistance exercise to modulate the glycaemic response. The apparent lack of glucose response following RE might be exacerbated by increased plasma glucose following HIE, which is consistent with findings of elevated glucose and insulin responses following high-intensity interval exercise completed 60 min after a breakfast meal [24]. Although the nutritional status of participants in the current study (all exercised in a fasting condition) may alter the known transient glucose response, it provides a very clear comparison of the two exercise modes. In the only trial known to evaluate exercise in a fasted condition, the post-prandial glucose response to the meal consumed one-hour after exercise was reduced more than exercising in a post-prandial state in people with type 2 diabetes [25]. While post-prandial glucose is important, the previous study [25] did not consider the immediate post-exercise phase in the fasted intervention, nor did it consider resistance exercise. Further research is therefore required to elucidate if the same response to HIE and RE occurs following exercise in a post-prandial state. Regardless, these results provide important information regarding exercise selection for individuals who typically rise early and exercise before consuming breakfast with the intent to improve their glucose regulation (for example, people with prediabetes). To induce glucose utilisation without risking a hyperglycaemic event, resistance exercise appears to be the preferred exercise modality.

A difference in physiological response was observed in the current study, through the elevation of cortisol with HIE. Cortisol secretion is associated with exercise intensity [26] and can impair peripheral glucose uptake, as well as stimulate hepatic glucose production [27]. Cortisol was significantly elevated by HIE in the current study, but not by RE. The difference in cortisol response to RE and HIE contrasts with previous findings comparing resistance exercise and high-intensity interval exercise matched for duration, but not exercise intensity in male and female adolescents [28]. The greater physiological stress with HIE could be induced by a larger requirement for energy transfer and number of muscle contractions required with high-intensity interval exercise. Muscle contraction and energy transfer are, in part, regulated by cAMP and calcium, which have been demonstrated to stimulate insulin secretion by augmenting the glucose stimulated upregulation of tryptophan hydroxylase 1 (Tph1), at least in rats [29]. Glucose can also induce rapid changes in calcium and cAMP signals within the β-cells of the pancreas [30], potentially stimulating further insulin secretion. It is not clear, in the current study, if differences in absolute workload (due to differences in physical capacity) might have contributed to different calcium secretion and signalling pathway activation; thus, this warrants further investigation. It is possible that this might also account for the increased blood lactate observed with HIE, indicating a larger contribution from the anaerobic energy system. Potentially, differences in the physiological response both directly and indirectly contribute to increases in glucose concentration through impaired glucose uptake and increased glucose production.

Although the HIE and RE bouts were prescribed at the same relative intensity with the same work and rest intervals, there were large differences in both heart rate and lactate responses. Previously, heart rate and energy expenditure have been elevated with kettlebell resistance exercise, compared to sprint interval cycling, when a greater period of work was completed with kettlebell exercise [19]. In comparison, adolescents completing high-intensity interval exercise experienced greater oxygen uptake than in the duration-matched resistance-exercise intervention [28]. There is a known linear relationship between heart rate and oxygen uptake; therefore, with the greater lactate response, we can only assume a greater energy requirement for HIE, as compared to RE, in the current study. This is despite the resistance exercises being chosen to work similar muscle groups that would be worked during the HIE and for the same duration.

The current investigation addressed several limitations of the existing literature relating to exercise duration and muscle groups. The collection of blood every thirty minutes allows for the acute response to be evaluated, to provide a greater level of understanding of glucose regulation. However, some limitations within this investigation need to be considered when interpreting the findings. Firstly, exercise was performed in a fasted state. Although some individuals might exercise in this condition, it is likely (but unknown) that most will exercise during the post-prandial or post-absorptive period, and we are aware of only one study specifically investigating a metabolic response to exercise implemented under this condition [25]. Several instances of nausea and presyncope were reported during both HIE and RE conditions that could be attributed to exercising in the fasted state. However, there was only one instance where this was severe enough to prevent the participant from completing the trial (completed 8 of the 12 HIE efforts). The lack of specific dietary control might also mean that gluco-regulatory responses could have been influenced by poor nutrition in the days leading up to each exercise bout. Secondly, oxygen consumption was not measured throughout each trial to confirm whether the matched relative exercise intensity equated to similar physiological work. While participants in the current study were recreationally active, they were not actively participating in the precise exercise protocols, as has occurred previously [28]. Although participants regularly completed high-intensity exercise, few were cyclists, and this might have contributed to the results. Finally, the participants recruited were all young, healthy individuals, and while this allows for a clear assessment of physiological responses, inflammatory/immunological responses that contribute to glucose regulation in disease states were not determined. There are also reports within the literature that menstrual-cycle phase will influence carbohydrate and fat metabolism; however, this is not conclusive with suggestions of within-phase variation and no difference in metabolic responses between different menstrual-cycle phases [31,32]. As we did not control for menstrual-cycle phase in female participants, this could be considered a limitation of our study; however, we expect that this would contribute only small differences if at all. Despite these factors, a clear difference in physiological response to the different modes of exercise has been identified. Further research is required to determine if these responses exist beyond the two-hour post-exercise period or if ongoing exercise training magnifies these transient responses to inform exercise guidelines.

## 5. Conclusions

In conclusion, resistance exercise appears to present itself as an alternative time-efficient exercise modality that limits a rise in blood glucose immediately after exercise in healthy adults. Resistance exercise resulted in lower glucose concentrations immediately after and up to 30 min following exercise, when compared to high-intensity interval exercise, despite a similar level of perceived effort. Preventing the acute elevation of glucose is an important consideration when prescribing exercise to those at risk of or with established cardiometabolic disease because of the known negative health consequences (micro- and macro-vascular disease). Resistance exercise has the potential to minimise the glucose elevations that are observed following high-intensity interval exercise.

## Figures and Tables

**Table 1:** Participant characteristics (Mean ± SD).

Variable	Overall (*n* = 16)	Male (*n* = 8)	Female (*n* = 8)	*p*-Value
Age (years)	22.4 ± 6.6	23.6 ± 9.5	21.1 ± 1.2	0.471
Height (cm)	173.49 ± 8.50	179.96 ± 4.89	167.01 ± 6.64	<0.001
Mass (kg)	70.71 ± 8.20	76.14 ± 4.96	65.30 ± 7.23	0.004
Peak Power (W)	302 ± 88	347 ± 100	257 ± 44	0.035
VO_2*peak*_ (mL∙kg∙min^−1^)	54.6 ± 10.9	57.6 ± 13.8	51.6 ± 6.5	0.284
1-RM Squat (kg)	100.1 ± 28.5	116.1 ± 29.7	85.7 ± 18.0	0.027
1-RM Calf Raise (kg)	179.6 ± 36.0	206.5 ± 30.8	152.8 ± 13.3	<0.001
1-RM Leg Press (kg)	187.7 ± 67.2	223.2 ± 70.3	152.3 ± 43.0	0.029

Peak power was calculated from a progressive cycle ergometer test to exhaustion, with peak power used to estimate peak oxygen uptake (VO_2*peak*_). A three-repetition maximum test was conducted for squat, calf raise and leg press exercises, with the 1-RM estimated by using the Brzycki method. The *p*-value is a comparison of male and female participants.

**Table 2:** Pre-exercise trial physiological variables (Mean ± SD).

Variable	Pre HIE	Pre RE	*p* Value
Glucose (mmol∙L^−1^)	4.4 ± 0.7	4.1 ± 0.8	0.070
Insulin (pmol∙L^−1^)	34.5 ± 11.5	52.7 ± 47.9	0.152
Cortisol (ng∙mL^−1^)	252.7 ± 77.3	239.2 ± 84.8	0.434
FFA (µM∙L^−1^)	146.7 ± 94.2	128.0 ± 125.8	0.631
Lactate (mmol∙L^−1^)	1.3 ± 0.4	1.8 ± 0.6	0.002
Heart rate (bpm)	80 ± 24	76 ± 15	0.722

HIE = high-intensity interval exercise; RE = resistance exercise; FFA = free fatty acids; bpm = beats per minute.

## Data Availability

The data presented in this study are available on request from the corresponding author. The data are not publicly available due to ethical approvals at the time.
